# Exosomes in arteriovenous fistula stenosis

**DOI:** 10.3389/fcell.2025.1663973

**Published:** 2025-09-19

**Authors:** Yushi Cao, Fangxiao Guo, Dandan Chen, Lei Li, Xiangyu Jie, Weitie Wang

**Affiliations:** ^1^ School of Biological Science and Medical Engineering, Beihang University, Beijing, China; ^2^ School of Clinical Medicine, Sun Yat-sen University, Shenzhen, Guangdong, China; ^3^ Department of Breast and Thyroid Surgery of Changchun Tumor Hospital, Changchun, Jijlin, China; ^4^ Department of Vascular Surgery, Qianwei Hospital of Jilin Province, Changchun, Jilin, China; ^5^ Department of Cardiovascular Surgery of the Second Hospital of Jilin University, Changchun, Jilin, China

**Keywords:** exosomes, arteriovenous fistula (AVF), extracellular vesicles, intimal hyperplasia, endothelial-to-mesenchymal transition (EndoMT), vascular remodeling, chronic kidney disease (CKD)

## Abstract

Arteriovenous fistula (AVF) stenosis is a complex pathological process caused by venous intimal hyperplasia, and its development is influenced by factors such as surgical injury, hemodynamic changes, inflammatory responses, and cellular proliferation and migration. Exosomes are critical mediators of intercellular communication and carry biomolecules (e.g., deoxyribonucleic acid, ribonucleic acid [RNA], and proteins) that can regulate cell functions and impact inflammatory responses, endothelial cell proliferation, and vascular smooth muscle cell migration. Studies have shown that molecules such as microRNAs within exosomes play significant roles in vascular stenosis-related diseases and can function as potential therapeutic tools and biomarkers for disease diagnosis. In addition, exosomes can serve as drug carriers with good biocompatibility and targeting capabilities, providing new avenues for the diagnosis and treatment of AVF stenosis. This article reviews the application of exosomes in AVF stenosis.

## 1 Introduction

Arteriovenous fistulas (AVFs) are the preferred vascular access for hemodialysis in patients with end-stage renal disease (ESRD). However, their long-term patency is often compromised by venous stenosis, primarily driven by neointimal hyperplasia, endothelial dysfunction, and vascular remodeling (Roy-Chaudhury et al., 2003). Traditional treatments such as percutaneous transluminal angioplasty (PTA) are limited by high restenosis rates and lack of long-term efficacy (Lee and Roy-Chaudhury, 2009).

In recent years, exosomes—nanosized extracellular vesicles released by most cell types—have attracted growing interest due to their ability to mediate intercellular communication via their cargo, including miRNAs, proteins, and lipids (Kalluri and LeBleu, 2020). A growing body of research indicates that exosomes regulate key cellular events implicated in AVF failure, such as vascular smooth muscle cell (VSMC) phenotypic switching, endothelial-to-mesenchymal transition (EndoMT), and chronic inflammation (Yanez-Mo et al., 2015).

This review aims to summarize the current knowledge on the biological functions of exosomes and their involvement in AVF stenosis, with a focus on their potential as diagnostic biomarkers and therapeutic delivery systems.

## 2 Exosomes: overview and biomedical relevance

Exosomes are nanosized extracellular vesicles (30–150 nm) secreted by most cells, playing critical roles in intercellular communication. They are formed via the endosomal pathway, in which multivesicular bodies (MVBs) release exosomes into the extracellular environment (Ludwig and Giebel, 2012). These vesicles carry various bioactive molecules such as proteins, lipids, DNA, and RNAs (including miRNAs, lncRNAs, and circRNAs), enabling them to influence recipient cell behavior (Robbin et al., 2014). Exosomes can cross biological barriers and deliver their cargo to recipient cells through endocytosis, membrane fusion, or receptor-ligand interactions.

Beyond their cargo composition, an essential property of exosomes lies in their intrinsic targeting ability. Exosomes exhibit selective tropism toward recipient cells depending on their membrane surface molecules, such as integrins, tetraspanins (CD9, CD63, CD81), and adhesion molecules. These molecules facilitate the interaction with specific receptors on target cells—including endothelial cells (ECs), vascular smooth muscle cells (VSMCs), macrophages, and fibroblasts—thereby determining tissue-specific delivery. Upon reaching the target, exosomes are internalized via multiple pathways, including clathrin-mediated endocytosis, macropinocytosis, and lipid raft–mediated fusion. This targeted delivery system enables exosomes to transfer functional RNA and protein payloads with high specificity and biocompatibility, making them promising candidates for vascular therapy and precision diagnostics (Ka et al., 2020; Yanez-Mo et al., 2015).

### 2.1 Biogenesis and target cell uptake

**FIGURE 1:**
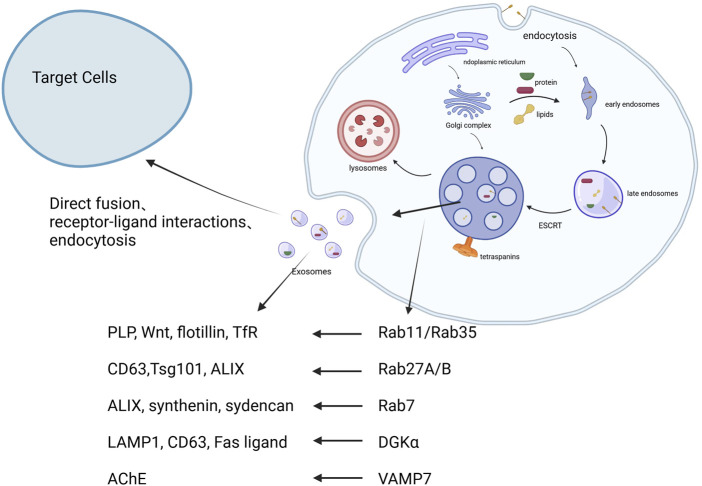
The biogenesis, release, and interactions with target cells of exosomes. 1. Early endosomes are formed through endocytosis. 2. These develop into multivesicular bodies (MVBs), regulated by ESCRT and tetraspanins. 3. MVBs fuse with lysosomes or the plasma membrane, releasing exosomes. 4. Exosomes interact with recipient cells via endocytosis, receptor-ligand interaction, or fusion Created in BioRender.com.

Exosome biogenesis involves invagination of the plasma membrane to form early sorting endosomes, which mature into late endosomes and MVBs (Ka et al., 2020). The sorting of cargo into intraluminal vesicles occurs through both ESCRT-dependent and independent mechanisms (van Niel et al., 2018). MVBs then fuse with the plasma membrane to release exosomes. Once secreted, exosomes interact with recipient cells via endocytosis, receptor-mediated uptake, or direct membrane fusion. These processes are regulated by proteins such as Rab GTPases, tetraspanins, and various cytoskeletal elements (Thery et al., 2018). The biogenesis, release, and interactions with target cells of exosomes are showed in Figure 1.

### 2.2 The composition of exosomes

**FIGURE 2:**
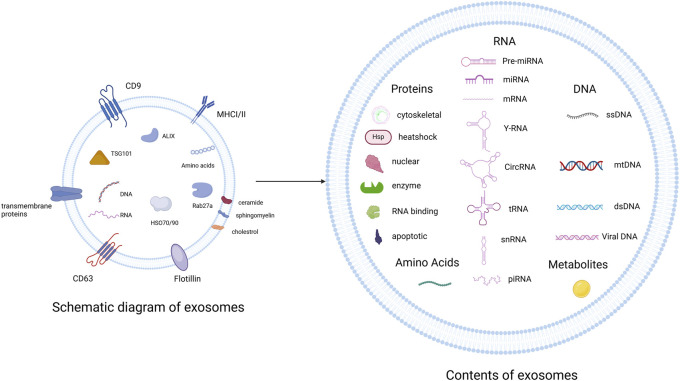
Exosomes are enriched with transmembrane proteins (CD9, CD63), MHC class I/II, TSG101/ALIX (ESCRT), HSP70/90, and various lipids (ceramide, sphingomyelin). Their cargo includes proteins, RNA species, DNA, and metabolites Created in BioRender.com.

Exosomes contain a lipid bilayer that protects their internal cargo. They are enriched with proteins such as CD9, CD63, and TSG101; lipids such as ceramide and cholesterol; and nucleic acids including miRNAs, lncRNAs, and circRNAs (Skog et al., 2008; Figure 2). These molecules mediate their functional effects. For example, miR-21, miR-146a, and miR-145 have been implicated in inflammation and vascular remodeling. lncRNAs and circRNAs contribute to transcriptional and post-transcriptional regulation (Valadi et al., 2007; Jiang N. et al., 2019).

#### 2.2.1 Proteins

The proteins within exosomes exhibit diverse functions and participate in various cellular processes such as cell adhesion, membrane fusion, signal transduction, and fundamental metabolism (He et al., 2018). Regardless of the cell type that secretes them, exosomes are rich in proteins and are highly stable owing to the protection offered by their bilayer lipid membranes. Flotillin-1, a lipid raft scaffold protein, is a nonspecific exosomal protein, involved in membrane fusion and transport (Kalluri and LeBleu, 2020). Rab GTPases control membrane and vesicle budding by recruiting effector proteins that play crucial roles in membrane fusion (Zhan et al., 2023). Heat shock proteins, such as the 70-kDa heat shock protein, can also be encapsulated in exosomes and primarily function in protein folding, refolding, and maintenance of protein homeostasis (Stenmark, 2009). Exosomes also contain major histocompatibility complex class I and II proteins, suggesting their potential for development as cancer vaccines owing to this characteristic. Exosomes derived from bodily fluids, such as blood, urine, and saliva, can be used as diagnostic biomarkers and prognostic indicators for cancer (Rosenzweig et al., 2019). A study employing highly rigorous isolation methods discovered multiple integrins and tetraspanins in exosomes that assisted in cell targeting and adhesion; these included CD9, CD63, CD81, and CD82 (Lyu et al., 2024). In contrast to the high abundance of these proteins, organelle-specific proteins from the Golgi apparatus and mitochondria are present in lower quantities in exosomes. Although different exosomal subpopulations may have varying protein profiles owing to environmental factors and different stimuli, there is no single marker that can be used to specifically identify exosomes (Pluchino and Smith, 2019; Yanez-Mo et al., 2015; Tauro et al., 2013).

#### 2.2.2 Lipids

Lipids are integral components of the exosomal membrane and contribute to the structural stability and fluidity of exosomes. They ensure the integrity of exosomes in body fluids and play crucial roles in exosome release. Owing to the presence of lipids, exosomes can fuse with the recipient cell membrane or be internalized by recipient cells, delivering their bioactive molecules to the target cells. The lipid composition of exosomes is not entirely identical to that of parent cells. Exosomes commonly contain lipids such as cholesterol, glycosphingolipids, sphingomyelin, ceramide, and phosphatidylinositol (Pallet et al., 2013; Skotland et al., 2019). The lipid composition of exosomes is similar to that of lipid rafts, exhibiting a higher lipid order and detergent resistance.

Some lipids in exosomes, which have not received much attention previously, also possess specific functions. For instance, ether lipids are involved in cell signaling and membrane transport (Skotland et al., 2020), whereas phosphatidylinositol phosphates participate in signal transduction and recruit proteins with specific recognition sites on the membrane (Dorninger et al., 2017). Phosphatidylserine (PS) and sphingomyelin on the exosomal membrane can bind to cell membrane receptors to initiate signaling pathways. For example, PS binds to phagocytic receptors, promotes exosome uptake, and influences immune responses (Nascimbeni et al., 2017). Lipids in circulating exosomes, particularly sphingosine and lysophosphatidylcholine, are closely associated with the development of hepatocellular carcinoma and can serve as potential biomarkers for the early detection of this disease.

#### 2.2.3 Nucleic acids

Nucleic acids are indispensable components of exosomes, playing a vital role as intermediaries in intercellular communication, and hold promise as diagnostic and prognostic biomarkers (Record et al., 2014). Exosomes contain various types of RNA, which are incorporated through specific sorting mechanisms, and even include some RNAs that are absent in parent cells. This allows cells to transfer RNA to other cells and tissues, thereby exerting functional effects (Kim et al., 2017). Additionally, some mRNAs are preferentially present in EVs and have been shown to translate into proteins at target locations using fluorescence methods (Skog et al., 2008). Another study demonstrated that miRNAs produced by EBV-infected cells can be transferred to uninfected cells to exert functional effects (Pegtel and Gould, 2019), confirming the functional transfer capability of exosomes and their nucleic acid content.

Exosomes encompass specific RNA subgroups rich in miRNAs, lncRNAs, and circRNAs. miRNAs have been extensively studied as endogenous non-coding nucleotides that post-transcriptionally regulate gene expression. Certain proteins recognize specific binding motifs of miRNAs and load them into exosomes (Skog et al., 2008). The miRNA profile of exosomes determines their cellular origin and influences the specificity of target organ uptake. miRNAs are associated with tumors and numerous diseases. For instance, miR-21-5p and miR-125-3p are linked to the pathogenesis of diabetic kidney disease, participating in cellular fibrosis and extracellular matrix accumulation (Pegtel et al., 2010). MiR-210 is related to TIMP-1 expression in lung adenocarcinoma cells, promoting tube formation activity in human umbilical vein endothelial cells (HUVECs) and accelerating lung adenocarcinoma metastasis (Villarroya-Beltri et al., 2013). miR-877 is involved in the induction of EBV-related tumors, while miR-21 and miR-29a bind to toll-like receptors (TLRs) in immune cells, triggering pro-metastatic inflammatory responses that lead to tumor growth and metastasis (Assmann et al., 2018). Furthermore, exosomal miRNAs may serve as markers to study degenerative conditions in humans. A study using microarray analysis to investigate the age-dependent differential expression of miRNAs found that although some highly abundant miRNAs did not exhibit differential expression, stratified analysis identified certain differentially expressed miRNAs (Cui et al., 2015). Another study screened and detected several miRNAs in saliva samples collected from subjects of different ages and found that salivary exosomal miRNAs provided a simple and effective means of measuring and assessing the aging process (Fabbri et al., 2012).

lncRNA are RNA molecules longer than 200 nucleotides that, despite not encoding proteins, play crucial roles in regulating gene expression. The structural characteristics of lncRNAs allow them to form various secondary and tertiary structures such as stem loops, pseudoknots, and triple helices. These structural features enable lncRNAs to interact with multiple molecules, including DNA, RNA, and proteins, thereby regulating gene expression and cellular functions (Tietje et al., 2014). LncRNAs possess specific domains that can fold into complex three-dimensional shapes. For example, Lnc-Nr6a1 mediates the assembly of the glycolysis complex by acting as a scaffold molecule for glycolytic enzymes, utilizing its structural domains (Machida et al., 2015). LncRNAs perform numerous functions in various biological processes. They regulate gene transcription and translation by interacting with transcription factors, chromatin-modifying enzymes, and ribosomes. Ye et al. discovered that the lncRNA KCNQ1OT1 interacts with the IκBα protein, reducing its phosphorylation, promoting IκBα ubiquitination, and sequestering miR-221, thereby increasing IκBα levels. Through this mechanism, KCNQ1OT1 inhibits inflammation and proliferation of vascular smooth muscle cells (VSMCs), reducing intimal hyperplasia (IH) (Zhang et al., 2024). lncRNAs can also act as signaling molecules, decoys, or guide molecules in various biological processes. Fan et al. identified lncRNA-XLOC_098131 as a novel lncRNA with immunoregulatory functions that acts as a competitive endogenous RNA to regulate toll-like receptor signaling pathways and immune functions (Polo-Generelo et al., 2022). Furthermore, lncRNAs play a critical role in cell differentiation. Qin et al. found that lncRNAs are involved in the regulation of muscle and adipose tissue differentiation via various molecular mechanisms in the nucleus and cytoplasm (Ye et al., 2020).

In recent years, circRNAs have become a significant research focus. CircRNAs are non-coding RNAs with a circular structure that lack a polyadenylated tail at the 3′end and a cap structure at the 5′end. This unique structure renders them resistant to exonucleases and provides high stability and abundant presence. Consequently, circRNAs degrade slowly and have been detected in blood, urine, the liver, and the pancreas, making them excellent biomarkers (Fan et al., 2019). circRNAs are formed by back-splicing when the spliceosome assembles on long exons, catalyzed by exon definition complexes (Yang et al., 2018). They can serve as templates for proteins and peptides. During translation, they directly bind to initiation factors. In addition, circRNAs often act as sponges for miRNAs or proteins and participate in the regulation of gene transcription. CircRNAs have been implicated in the pathophysiology of various tumors (Li et al., 2015), influencing glycolysis, lipid metabolism, and other energy metabolism processes by regulating transport proteins, transcription factors, and signaling pathways. These processes play crucial roles in tumor development (Li et al., 2019). CircRNAs also regulate the epithelial–mesenchymal transition process, contributing to tumor proliferation and metastasis. In addition to oncology, circRNAs are involved in other diseases, such as osteoarthritis, cardiovascular diseases, and degenerative conditions (Tang et al., 2022).

### 2.3 Target cell uptake

After exosomes are secreted, they are taken up by target cells through several distinct mechanisms. Endocytosis is the primary method of exosome uptake and is mediated by various substances including lipid rafts, clathrin, and caveolin. For example, exosomes derived from Schwann cells enter target cells via lipid raft-mediated endocytosis and regulate macrophage polarization via the SOCS3/STAT3 pathway to alleviate inflammatory responses (Yu et al., 2019). Additionally, the primary mechanism for exosome-like vesicle uptake from *Opisthorchis felineus* by human cholangiocytes is clathrin-mediated endocytosis, highlighting the significance of different endocytic pathways in various cell types (Wang et al., 2019). Myriam et al. reported a unique pathway for exosome uptake in hypoxic environments. They discovered that under hypoxic conditions, heparan sulfate proteoglycan-dependent endocytosis promotes exosome uptake, leading to lipid droplet formation in glioma cells (Ren et al., 2023). Exosomes are preferentially taken up by homologous cells through receptor-ligand interactions, making them highly likely to be enclosed by lysosomes. Shabirul et al. designed exosomes to express CD19 chimeric antigen receptors, enabling them to target CD19-positive leukemia B cells through receptor-ligand interactions and induce cell death without affecting CD19-negative cells (Pakharukova et al., 2023). Membrane fusion is a common mechanism for heterologous cell uptake. Exosomal contents are delivered to the target cell when the exosomal membrane fuses with the plasma membrane, and the exosomal contents are then delivered into the target cell (Cerezo-Magana et al., 2021). Exosomes secreted by dendritic cells can fuse with target cells, creating fusion pores that release their contents (Haque et al., 2021). The uptake of exosomes by target cells is influenced by the specific surface proteins of both the exosomes and target cells, as well as the external environment. Plasma proteins attached to the surfaces of exosomes can alter their functional properties, provide new functionalities, and affect their uptake by target cells (Montecalvo et al., 2012). Lower pH levels in the environment significantly affect EV secretion and increase EV uptake (Chernomordik and Kozlov, 2008).

### 2.4 Isolation and identification methods

**FIGURE 3:**
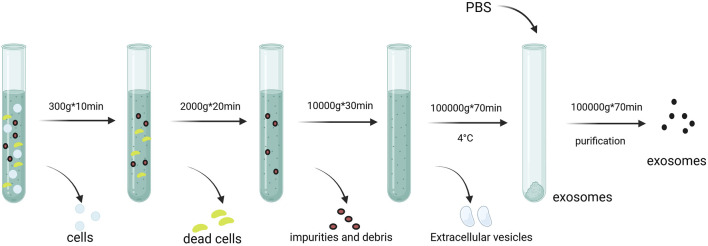
Isolation and extraction of exosomes. Centrigugal speeds of 300*g, 2000*g, and 10,000*g are used to remove impurities and debris. Exosomes are finally obtained by 100,000*g speed Created in BioRender.com.

Common exosome isolation techniques include ultracentrifugation (Figure 3), PEG precipitation, and size-exclusion chromatography (Konoshenko et al., 2018). Immunoaffinity capture and commercial kits like ExoQuick are also used. Characterization methods include nanoparticle tracking analysis (NTA), transmission electron microscopy (TEM), and flow cytometry (Table 1). While each technique has advantages and drawbacks, combining approaches improves yield and purity (Zhang et al., 2016). Detailed reviews on methodology are available for further reference (Mateescu et al., 2017).

**TABLE 1:** Current methods for identification of exosomes.

Method	Advantages	Disadvantages
Flow cytometry	Capable of analyzing a large number of samples for high-throughput analysis Able to simultaneously detect multiple surface markers Can detect very low concentrations of exosomes through fluorescent labeling	Presence of impurities in the sample may affect the accuracy and specificity of the results Requires high standards for instrumentation and operational technique, increasing cost and complexity
Transmission electron microscopy	Extremely high resolution, enabling clear observation of exosome details and even internal structures and components Can provide three-dimensional structural information Using immunogold labeling technology, can analyze internal specific proteins and provide functional information	Sample preparation is complex and time-consuming, requiring skilled technicians Negative staining and drying steps may introduce artifacts, affecting morphological judgment Equipment is expensive, limiting its application for large-scale sample analysis
Dynamic light scattering	Simple sample preparation Capable of analyzing a large number of samples in a short time Does not damage the sample structure, maintaining its natural state	Difficulty in distinguishing particles of similar sizes Requires high sample purity Can only measure size and distribution, unable to provide morphological information
Nanoparticle tracking analysis	Can measure individual particles, providing high-resolution size information Capable of quantitative analysis of particle concentration in the sample Simple sample preparation and quick operation	Requires very high sample purity Limited resolution for extremely small particles (<20 nm) or very large particles (>1 µm) Data processing is complex

### 2.5 Storage of exosomes

Exosomes are a focal point of research in the fields of medicine and biomedicine, and optimizing their preservation is a prerequisite for further study. Research indicates that the storage methods of exosomes significantly impact their concentration, physical state, and biological functionality (Jeyaram and Jay, 2017). Therefore, optimizing storage conditions is crucial for the reliability of fundamental research results on exosomes, the efficacy of clinical applications, and the quality and safety of commercialized products. Currently, storage at −80°C is a widely adopted method. Stefano et al. systematically evaluated eight different strategies, including simple −80°C freezing and the addition of seven different protective agents, as well as the effects of freeze-thaw cycles on exosomes. The results indicated a negative correlation between storage time and the concentration and purity of exosomes, with particle size and variability changing over time. They recommended that, in most cases, exosomes should be sourced from fresh samples. For strict storage requirements, biological samples may be stored short-term, while storing isolated exosomes is not advised (Gelibter et al., 2022). Yuan et al. proposed that storage at 4°C can be utilized for short-term preservation of exosomes. However, for long-term retention of functionality and extended shelf life, freeze-drying presents a promising method (Yuan et al., 2021). André et al. found that storing exosomes in phosphate-buffered saline (PBS) leads to a significant decrease in recovery rates, particularly for pure samples. They applied a novel buffer, which is a PBS formulation supplemented with human albumin and trehalose (PBS-HAT), and discovered that it significantly enhances both the short-term and long-term preservation of exosomes, especially under −80°C conditions (Gorgens et al., 2022). Further research is essential to explore optimal methods for the long-term or short-term preservation of exosomes while maintaining their inherent properties and functionalities.

### 2.6 Functions of exosomes

Almost all human cells secrete exosomes that fuse with target cells to transfer functional molecules or activate receptors or effectors. Exosomes released through various pathways can act as signaling messengers, regulating the behavior of nearby, distant, or even originating cells. For instance, hepatocyte-derived exosomes mediate increased synthesis of sphingosine-1-phosphate by transferring neutral ceramidase and sphingosine kinase 2, thereby promoting hepatocyte proliferation and regeneration following ischemia-reperfusion injury or partial hepatectomy (Wu and He, 2023). Exosomes released by ECs under oxidative stress conditions can enhance EC proliferation and migration and transport miR-92a-3p to VSMCs, promoting their proliferation and migration (Guo and Zhang, 2023). Using a rat model, Qu et al. demonstrated that exosomes derived from human umbilical cord mesenchymal stem cells (hucMSCs) enhanced endothelial function, inhibited neointimal hyperplasia in vein grafts, and accelerated reendothelialization (Nojima et al., 2016). These exosomes promoted EC proliferation and migration, thereby contributing to vascular repair and regeneration.

Exosomes play crucial roles in cellular immune responses. Certain exosomes secreted by macrophages can induce proinflammatory reactions. For instance, exosomes containing glycopeptidolipids from *Mycoplasma gallisepticum*-infected macrophages can stimulate resting cells to exert proinflammatory responses (Yao et al., 2022). Under stress conditions, heat shock proteins are embedded in the plasma membrane and then released into the environment, inducing tumor necrosis factor alpha (TNF-α) release and activating macrophages (Qu et al., 2020). Neutrophil-derived exosomes transfer miR-30d-5p, inducing M1 macrophage polarization and activating the nuclear factor kappa B (NF-κB) signaling pathway, leading to an inflammatory response in macrophages (Bhatnagar and Schorey, 2007). Zhang et al. studied the effects of exosomes derived from *Treponema pallidum*-stimulated dendritic cells on EC inflammation. These exosomes activated the LR4/MyD88/NF-κB signaling pathway, increasing the expression of interleukin (IL)-1β, IL-6, and TNF-α in ECs, thereby promoting inflammation. Plasma exosomes from patients with acute myocardial infarction can induce NF-κB signaling activation, contributing to proinflammatory immune responses and EC injury (Vega et al., 2008). Moreover, exosomes can modulate the tumor microenvironment and enhance or reduce the responsiveness of cancer cells to therapies. They transport non-coding RNAs and regulate molecular signaling pathways such as PTEN and PI3K/Akt, promoting chronic inflammation, immune evasion, and tumor progression (Hazrati et al., 2022).

Exosomes play a significant role in neuronal signal transmission, promoting neuronal survival and synaptic assembly. They can cross the blood–brain barrier and are involved in myelination. Exosomes are associated with the pathological processes of neurodegenerative diseases, such as Alzheimer’s disease, Parkinson’s disease, multiple sclerosis, amyotrophic lateral sclerosis, and Huntington’s disease (Giannotta et al., 2013). Aging is regulated by the hypothalamus. A study involving mice revealed that exosomes secreted by normal hypothalamic stem cells can slow aging, suggesting that the hypothalamus partially controls this process through the release of exosomes (Gong et al., 2023).

## 3 Arteriovenous fistula stenosis

**FIGURE 4:**
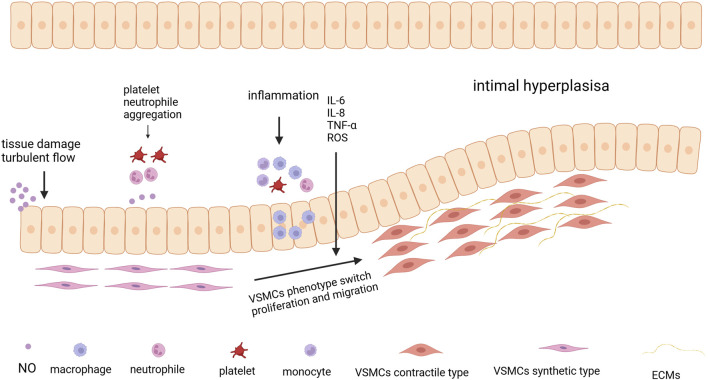
The process of Intimal Hyperplasia. The process begins with tissue damage and turbulent blood flow, which can injure the endothelial cells lining the blood vessel. Following endothelial injury, platelets and neutrophils aggregate at the site of damage. Then the aggregation of immune cells, such as monocytes and macrophages, leads to the release of pro-inflammatory cytokines like IL-6, IL-8, TNF-α, and reactive oxygen species (ROS) exacerbating the inflammatory response. Under the influence of inflammatory signals, VSMCs undergo a phenotypic switch from a contractile type to a synthetic type Created in BioRender.com.

The increasing aging population and prevalence of chronic diseases have led to an increased incidence of end-stage renal disease. Well-functioning hemodialysis access is crucial for the survival of patients with kidney disease. An autogenous arteriovenous fistula (AVF) is the first clinical choice for dialysis access; however, dysfunction of the dialysis pathway often leads to repeated surgeries and even patient mortality. Venous IH is the primary cause of dialysis access failure. The factors contributing to IH are categorized as upstream and downstream events. Upstream events include endothelial injury caused by surgical trauma and hemodynamic changes, whereas downstream events include oxidative stress response, inflammatory response, cell proliferation, and migration (Cheng et al., 2023). (Figure 4.) IH is a complex pathophysiological process involving numerous cells and signaling pathways. For instance, endothelial cell injury leads to a weakened barrier function of the vascular wall, the release of growth factors such as PDGF, and the secretion of pro-inflammatory factors that activate inflammatory pathways. Ultimately, this results in the proliferation and migration of smooth muscle cells. These smooth muscle cells migrate from the media to the intima and proliferate, forming a thickened intimal layer, which consequently causes vascular narrowing. Therefore, a detailed understanding of the migratory and transformed cellular components and molecular pathways involved is essential for developing targeted therapies in the future.

### 3.1 Tissue injury

Surgical procedures and dialysis punctures can cause tissue injury, and EC damage is particularly significant. Under physiological conditions, ECs form a continuous endothelium that lines the inner walls of blood vessels. This endothelium not only facilitates the metabolic exchange of plasma and tissue fluid but also secretes various bioactive substances that maintain vascular tone, regulate blood pressure, and balance antiinflammatory and proinflammatory responses. Compromised integrity of the endothelium leads to the invasion of inflammatory cells, platelet aggregation, and thrombosis (Galardi et al., 2023). Oxidative stress is a crucial mechanism underlying endothelial damage. During oxidative stress, the balance between oxidation and antioxidation is disrupted owing to the excessive production of reactive oxygen species (ROS). Hypoxia and substances, such as angiotensin II, can induce ROS production, leading to endothelial tissue damage (Lee and Roy-Chaudhury, 2009). ROS reduces the bioavailability of nitric oxide (NO) through redox reactions, causing vascular dysfunction. NO plays a crucial role in the cardiovascular system by relaxing vascular smooth muscle, promoting vasodilation, and enhancing blood flow. Additionally, it possesses anti-inflammatory properties, supports endothelial cell function, and inhibits platelet aggregation. Zhu et al. designed a patch that covalently binds a nitrite compound to a biodegradable polymer for localized NO release. Their study, conducted in animal models, demonstrated that the released NO improves cardiac function, aids in cardiac tissue repair, and alleviates cardiac remodeling and fibrosis (Zhu et al., 2021). In another study, researchers developed a precise system for delivering nitric oxide (NO) to tissues, which demonstrated the critical role of NO in vascular regulation and tissue repair in both rat hind limb ischemia models and mouse acute kidney injury models (Hou et al., 2019). Furthermore, ROS can trigger endothelial inflammation, disrupt mitochondrial function, and lead to abnormal energy metabolism, exacerbating EC damage (Wettschureck et al., 2019). ROS can induce EC apoptosis by increasing the expression of p38MAPK and caspases and by inhibiting the expression of Bcl-2 while promoting the expression of Bax and Fas. It can also induce apoptosis in VSMCs (Shaw et al., 2014). ROS have been recognized as a significant target in ischemia-reperfusion injury. Li et al. developed a ROS-sensitive hydrogel for the delivery of basic fibroblast growth factor (bFGF) aimed at repairing damaged cardiomyocytes and enhancing angiogenesis (Zhenhua Li et al., 2021). The NLRP3 inflammasome activation plays a significant role in endothelial dysfunction (Shaito et al., 2022). The NLRP3 inflammasome can activate and promote the maturation of IL-1β and IL-18 and mediate the formation of the pore protein, N-GSDMD, leading to cell swelling, rupture, and death (Higashi, 2022). It can also disrupt tight junction proteins between ECs, thereby increasing vascular permeability. As an upstream signal of NLRP3, ROS initiates and activates the NLRP3 inflammasome, thereby triggering pyroptosis.

### 3.2 Hemodynamic changes

Blood flow in vessels can be categorized into laminar and turbulent flows based on different flow patterns, which are closely related to the vascular configuration. In smooth and straight sections of the lumen, blood flow is typically laminar, with a Reynolds number (Re) of less than 2000. Conversely, in irregular sections, including the inner sides of curved vessels and upstream stenotic regions, the flow becomes unstable and disturbed, manifesting as a turbulent flow with an Re greater than 2000. The original vascular anatomy is altered following AVF creation, resulting in different angles between the arteries and veins. This alters the type of shear stress acting on the endothelium, shifting it from laminar shear stress to low oscillatory shear stress (Bai B. et al., 2020). The EC membrane contains numerous receptors that detect and transmit mechanical stimuli, including receptor tyrosine kinases, G protein-coupled receptors, and various ion channels (Zhang Y. et al., 2019). This mechanosensory capability helps differentiate blood flow characteristics and make physiological adjustments.

Under laminar shear stress, the expression of the cyclin-dependent kinase inhibitor p21 increases, slowing EC DNA synthesis and inhibiting proliferation. Laminar shear stress can suppress apoptosis induced by serum starvation, oxidative stress, and TNFα, potentially through increased expression of nitric oxide synthase and superoxide dismutase (Pfenniger et al., 2015). NO produced by ECs can elevate cGMP and PKG levels, reducing Ca2+ concentration and promoting vasodilation (He et al., 2022). Under physiological conditions, laminar shear stress also reduces the expression of the adhesion molecules, vascular cell adhesion molecule one and intercellular adhesion molecule 1. This in turn decreases leukocyte and platelet aggregation, reduces ROS levels, activates anticoagulant genes, and inhibits VSMC proliferation and migration. These actions help maintain EC vitality and functional integrity (Rennier and Ji, 2013; Cirino et al., 2017). In contrast, under low shear stress, cell proliferation is accelerated, ROS levels are increased, and IL-6 and IL-8 secretion is significantly increased, activating neutrophils and promoting their adhesion to ECs, thereby fostering inflammation and apoptosis.

### 3.3 Inflammation

Inflammation is a key factor in IH development. The inflammatory response involves various miRNAs that target and regulate multiple proteins to promote inflammation; these include miR-125, miR-155, and miR-125. Following tissue injury, leukocytes aggregate at the injury site under the influence of chemokines (Zheng et al., 2013). Macrophages and neutrophils release a plethora of inflammatory cytokines such as interleukins, C-reactive protein, and TNF-α during the inflammatory response. These pro-inflammatory cytokines, in conjunction with growth factors, further promote the aggregation of inflammatory cells and initiate the phenotypic transformation of VSMCs, leading to their proliferation and migration (Wang et al., 2014). Chemokines that promote leukocyte aggregation, such as platelet-derived growth factor (PDGF) and tumor growth factor-β, are secreted by VSMCs and activated by ECs. Local inflammation also triggers vascular wall remodeling and neointimal formation, leading to vascular intima stenosis. Yi et al. discovered that Nik-related kinase expression in VSMCs affects IH and vascular inflammation by regulating the expression of matrix metalloproteinases and inflammatory cytokines/chemokines (Charo and Taubman, 2004). The epigenetic factor, PCAF, is also involved in regulating vascular inflammation by promoting NF-κB-mediated inflammation (Carracedo et al., 2019). Further, reduced PCAF levels significantly decreased IH, the intima/media ratio, and luminal stenosis. Xu et al. revealed that Sox10 is a key regulator of vascular inflammation (Lu et al., 2020). It activates the PI3K/AKT signaling pathway and drives the transdifferentiation of VSMCs into macrophages through lactylation and phosphorylation modifications, thereby promoting pyroptosis and exacerbating the inflammatory response.

### 3.4 Endothelial cells

Upon stimulation, ECs contract, leading to the disruption of adhesive junctions. Adhesion between ECs is primarily maintained by vascular endothelial cadherin (VE-cadherin), which interacts with cytoskeletal molecules to regulate EC adhesion, vascular regeneration, and migration (de Jong et al., 2017). VE-cadherin connects with β-catenin, plakoglobin, and p120-catenin to stabilize EC junctions (Xu et al., 2023). The barrier function of ECs is dynamic and maintained by adhesive junctions. Endothelial permeability increases under the influence of autocrine substances such as vascular endothelial growth factor (VEGF) and thrombin. This dynamic change during vascular injury promotes the accumulation of inflammatory mediators, leukocyte migration, and Ca2+ elevation and activates Ca2+-driven proteins such as myosin light chain kinase and membrane-associated protein A2. These processes promote actin contraction and the degradation of endothelial cadherin, leading to EC detachment and barrier dysfunction (Giannotta et al., 2013; Oas et al., 2013). Additionally, talin-dependent activation of EC β1-integrin weakens adhesion and reduces tyrosine phosphorylation of the VE-cadherin-catenin complex, and VEGF activation of FAK kinase opens endothelial junction structures (Dalal et al., 2020). Collectively, these processes contribute to the loss of endothelial function.

Endothelial–mesenchymal transition (EndoMT) refers to a pathological process in which endothelial cells lose their specific markers (e.g., CD31 and VE-cadherin) and acquire a mesenchymal phenotype characterized by the expression of α-SMA, vimentin, and other mesenchymal proteins. This transition is commonly induced by inflammatory cytokines, oxidative stress, or transforming growth factor-beta (TGF-β), and has been shown to play a pivotal role in vascular remodeling and neointimal hyperplasia, particularly following vascular injury (Qian et al., 2025). Emerging studies have indicated that exosomes also play critical roles in modulating EndoMT. Exosomes derived from diseased or inflamed tissues may promote EndoMT, whereas those from mesenchymal stem cells or other regenerative sources can inhibit EndoMT and preserve endothelial identity, offering potential therapeutic avenues for limiting intimal hyperplasia in AVF (Sishuai et al., 2024). Additionally, under oxidative stress conditions, exosomes derived from adipose-derived stem cells (ADSC-Exo) have been shown to alleviate hydrogen peroxide-induced EndoMT in human umbilical vein endothelial cells (HUVECs) by inhibiting the miR-486-3p/Sirt6/Smad signaling axis, suggesting their antifibrotic potential in endothelial dysfunction (Li et al., 2024).

During neointimal formation following vascular injury, ECs play a crucial role, with some cells undergoing competitive selection to become tip cells that rearrange dynamically to form new blood vessels (Pulous et al., 2019). In this process, the interaction between the Notch and YAP/TAZ signaling pathways regulate the selection and phenotype of tip cells (Chen et al., 2012). Chen et al. found that the transcription factor Foxp1 in ECs can regulate VSMC proliferation and migration by targeting the matrix metalloproteinase (MMP)-9 and Cdkn1b signaling pathways (Blanco and Gerhardt, 2013). When Foxp1 is absent, VSMC proliferation and migration increase, while endothelial cell proliferation decreases.

### 3.5 Vascular smooth muscle cells

VSMCs are abundant in the vasculature and primarily comprise the medial layer of blood vessels. The active contraction and passive recoil of elastin fibers contribute to the elasticity of the blood vessels, thereby regulating blood pressure and flow distribution. VSMCs exhibit high plasticity, phenotypic switching capabilities, and proliferative potential, which enable adaptive responses to environmental changes. They are regulated by many miRNAs (Table 2). Under normal physiological conditions, VSMCs express many contractile proteins such as myosin heavy chain 11, SM22α, and α smooth muscle actin, which confer a contractile phenotype (Guo et al., 2024). Their contraction activity is regulated by calcium ions, and calmodulin activates myosin light chain phosphorylation, leading to myofibril contraction. However, under various pathological conditions, VSMCs can switch from a contractile phenotype to a synthetic phenotype, aiding in injury repair. In the synthetic phenotype, the cells change from a spindle shape to an irregular shape, with a significant reduction in contractile markers. Their proliferation and migration abilities are enhanced, and they secrete large amounts of extracellular matrix, including collagen, elastin, and matrix metalloproteinases, in response to injury (Chen et al., 2022; Chakraborty et al., 2021). Phenotypic switching of VSMCs is typically regulated by inflammatory mediators, growth factors, transcription factors, and noncoding RNAs. For instance, growth factors can stimulate VSMC dedifferentiation through the MAPK pathway, whereas transcription factors such as KLF4 can regulate phenotypic switching by modulating epithelial adhesion complexes and actin cytoskeleton dynamics (Lynch et al., 2016; Jain et al., 2020). Synthetic VSMCs migrate from the medial layer to the intima, forming a new intimal layer that causes venous neointimal hyperplasia.

**TABLE 2:** Exosome-derived miRNAs involved in AVF stenosis and their molecular mechanisms.

miRNA	Pathway	Function	References
.miR-21	PTEN	Promotes VSMC proliferation and migration by inhibiting PTEN	Zhu et al. (2019)
miR-221	p27Kip1/cyclin-dependent kinase	Promotes cell cycle progression and VSMC proliferation by downregulating p27KIP1	Liu et al. (2009)
miR-222	p27Kip1/cyclin-dependent kinase	Promotes cell cycle progression and VSMC proliferation by downregulating p27KIP1	Liu et al. (2009)
miR-145	Smad4	Inhibits VSMC proliferation and migration	Li et al. (2018)
miR-146	TRAF6	Reduces VSMC proliferation, migration, inflammation, and ROS production by inhibiting TRAF6	Yang et al. (2022)
miR-155	AKT1	Inhibits VSMC proliferation and migration	Chen et al. (2019)

VSMC, vascular smooth muscles cell; ROS, reactive oxygen species; PDGFR-β, platelet-derived growth factor receptor beta.

### 3.6 Ferroptosis

**FIGURE 5:**
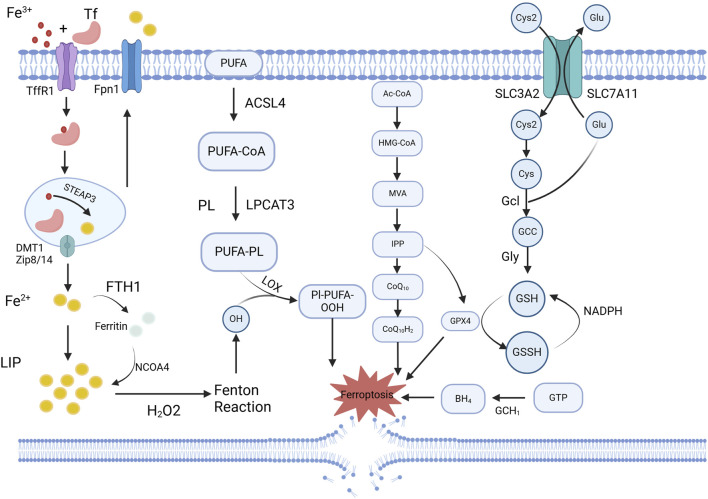
Schematic overview of ferroptosis-related mechanisms regulated by exosomal miRNAs. Ferroptosis is characterized by iron accumulation, lipid peroxidation, and impaired antioxidant defense, particularly through downregulation of GPX4 and system Xc^−^ (SLC7A11). Exosomal miRNAs, such as miR-27a-3p and miR-125b, modulate these pathways by targeting key regulators like SLC7A11, Keap1/Nrf2, or GPX4, thereby influencing ferroptotic sensitivity in vascular cells. Created in BioRender.com.

Ferroptosis, a research hotspot in recent years, is a form of programmed cell death that is dependent on iron and lipid peroxidation. It is primarily regulated by three major mechanisms—iron metabolism, lipid metabolism, and the glutathione peroxidase 4 (GPX4) pathway activation. Other pathways involved include FSP1-CoQ10-NAD(P)H, BH4-DHFR, and P53 signaling (Yoshida et al., 2012). (Figure 5.) Ferroptosis is closely associated with intimal vascular stenosis. Using differentially expressed gene analysis, Zhang et al. identified 34 and 31 significantly differentially expressed ferroptosis-related genes at 2 days and 14 days after carotid artery ligation in mice, respectively (Han et al., 2021). Exposure to ZnO nanoparticles (ZnONP) induces endothelial dysfunction via ferroptosis. When ZnONP exposure triggers vascular inflammation, ferroptosis markers increase, and the inhibition of ferroptosis reduces vascular damage (Zhang et al., 2021). Chen et al. found that the phospholipid oxidation product, PGPC, increases ferrous ion content and peroxide production, inducing EC ferroptosis via the CD36 receptor, thereby impairing endothelial function by increasing FABP3 expression (Zhang L. et al., 2022). Luo et al. discovered that ferroptosis is closely related to EC death, activating the p53-xCT-GSH axis and causing endothelial dysfunction (Qin et al., 2021). Zhang et al. found that BaP/BPDE exposure upregulates free iron, accelerates lipid peroxidation, and reduces GPX4 protein levels, thereby inducing ferroptosis, inhibiting EC function, and affecting angiogenesis (Chen et al., 2024).

Ferroptosis also induces VSMC phenotypic switching. *In vivo* activation of ferroptosis by RSL3 promotes VSMC phenotypic switching and exacerbates IH. A previous study showed that the ferroptosis inhibitor, Fer-1, counteracted the effects of RSL3 by reducing IH (Luo et al., 2021). Sun et al. discovered that ferroptosis promotes VSMC aging and vascular aging (Zhang et al., 2023). Activation of ferroptosis signaling led to a decrease in NAD + levels and drove VSMC aging via NCOA4-mediated ferritinophagy. Using a specific inhibitor of lysine-specific demethylase 1 has been found to reduce intracellular iron levels and decrease VSMC ferroptosis (Zhang S. et al., 2022). Targeted inhibition of the endogenous histone acetyltransferase P300 activates the hypoxia-inducible factor 1-alpha (HIF1-α)/HMOX1(Heme Oxygenase 1) axis, promoting CD and IKE-induced VSMC ferroptosis, thus contributing to vascular stenosis-related diseases. Ferroptosis is closely related to inflammation, which influences vascular intimal stenosis by modulating inflammation-related pathways. Chen et al. concluded that ferroptosis regulate the activity of five inflammatory signaling pathways—JAK-STAT, NF-κB, inflammasome, cGAS-STING, and MAPK, thereby affecting cellular functions (Sun et al., 2024).

## 4 Role of exosomes in arteriovenous fistula stenosis

**FIGURE 6:**
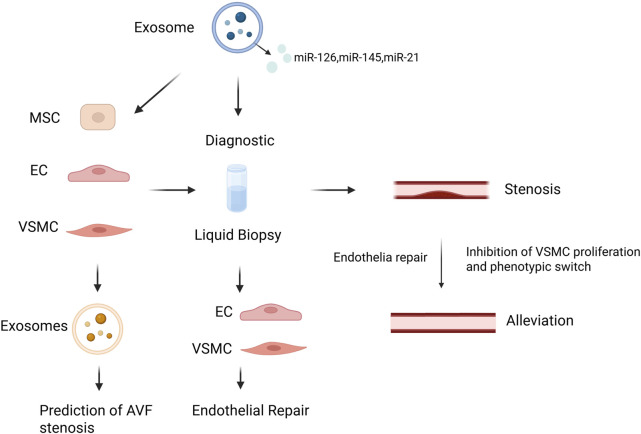
Schematic illustration of exosome-based diagnostic and therapeutic strategies in AVF stenosis. Created in BioRender.com.

Exosomes play a significant role in the progression of AVF stenosis by affecting EC function, inflammatory responses, and proliferation and migration of VSMCs (Figure 6). One of the causes of AVF stenosis is EC damage and dysfunction, which are responsible for regulating inflammation, vascular tone, and permeability. The damaged ECs are replaced by proliferating resident ECs, followed by endothelial apoptosis and necrosis. Exosomes are released when ECs are subjected to shear stress and injury, affecting NO release and altering vascular tone, further compromising endothelial function (He et al., 2024). Endothelial progenitor cells (EPCs) are a type of stem cells that serve as precursors to ECs, with strong growth and differentiation potential. Exosomes derived from EPCs contain various factors that regulate vascular function, promote neovascularization, and repair endothelial dysfunction (Chen et al., 2023; Lovren and Verma, 2013). For example, EPC-derived exosomes are enriched in angiogenic microRNAs such as miR-126, which enhances endothelial proliferation and migration via the PI3K/Akt pathway. Moreover, these exosomes may carry angiogenic proteins such as VEGF, promoting endothelial cell survival and tube formation, thereby facilitating vascular repair and new vessel formation (Jansen et al., 2012; Zhang et al., 2015). EPCs and ECs can influence phenotypic switching of VSMCs, thereby contributing to AVF stenosis. A recent study found that exosomes from EPCs adhere to damaged vascular areas, inhibit post-injury neointimal formation, reduce inflammation levels, and enhance endothelial function by antagonizing apoptosis via the Bcl2/Bax/Caspase-3 signaling pathway (Xing et al., 2020). Exosomes secreted by ECs contain miR-195, which can regulate VSMC proliferation, as well as proteins (e.g., PDGF-BB) that affect VSMCs (Bai S. et al., 2020; Tan et al., 2024). Another study demonstrated that EC-derived EVs also participate in regulating monocyte activation, thereby influencing vascular inflammation (Gu et al., 2017). Under the influence of angiotensin-converting enzyme 2, EPC-derived exosomes enhance mitochondrial function and regulate VSMC phenotype, thereby protecting against EC injury (Wang et al., 2020a).

Inflammation is a crucial pathological process leading to vascular stenosis, and exosomes play a significant role in this process. Exosomal miRNAs can regulate inflammatory responses. The expression profiles of exosomal miRNAs in patients with asymptomatic carotid stenosis are associated with post-endarterectomy inflammation and major adverse cardiovascular events (Togliatto et al., 2018). Proteins within exosomes such as MFGE8 promote the polarization of antiinflammatory M2b macrophages and reduce collagen deposition and inflammation. Animal experiments have shown that MFGE8 prevents esophageal stenosis following endoscopic submucosal dissection (Wang et al., 2020b). Exosomes derived from MSCs have significant potential in reducing inflammation and promoting vascular healing. Adipose-derived MSCs overexpressing stanniocalcin-1 can inhibit NLRP3 inflammasome-mediated inflammation and promote reendothelialization in mechanically injured carotid arteries after endarterectomy (Sardu et al., 2021). Another study established a rat model of renal artery stenosis-induced kidney injury and found that adipose-derived stem cell exosomes reduced the expression of the hypoxia marker, HIF1-α and stabilized blood pressure, indicating their role in mitigating inflammation and vascular damage (Lai et al., 2024). Yang et al. discovered that EC-derived exosomes reduced neointimal hyperplasia resulting from carotid artery injury in rats by inhibiting the ROS-NLRP3 inflammasome and reducing VSMC phenotypic switching (Liu et al., 2022).

Exosomes have been found to influence the behavior of VSMCs and play significant roles in various pathophysiological processes. In atherosclerotic diseases, exosomes regulate the proliferation and migration of VSMCs via the circ-100696/miR-503-5p/PAPPA axis, thereby promoting the progression of atherosclerosis (Ishiy et al., 2020). Additionally, exosomes secreted by VSMCs in patients with diabetes enhance endothelial activation and inflammatory polarization of macrophages through miR-221/222, thereby promoting vascular inflammation and accelerating atherosclerotic plaque formation (Yang et al., 2023). A study verified whether miR-339-3p affects angiotensin II type-1 receptor autoantibody-induced vascular inflammation using an animal model. The results revealed that miR-339-3p in exosomes promotes inflammatory responses by upregulating the NFATc3 protein. This demonstrates that miRNAs in exosomes can modulate inflammatory responses in VSMCs and contribute to vascular stenosis (Liu et al., 2023). Furthermore, studies related to carotid artery stenosis have shown that lncRNA PCA3 inhibits the proliferation, migration, and invasion of VSMCs by negatively regulating the miR-124-3p/ITGB1 axis, providing valuable therapeutic targets for vascular stenosis-related diseases (Yu et al., 2023). Many other miRNAs are involved in the proliferation, migration, and phenotypic switch (Table 3).

**TABLE 3:** The role of miRNAs in ferroptosis.

MiRNA	Pathway	Function	References
miR-9	Erastin and RSL3	Inhibit ferroptosis by reducing lipid peroxidation and iron accumulation	Zhang et al. (2018)
miR-21	P53/SLC7A11	Inhibit ferroptosis by inhibiting p53 gene and protein expression	Zou et al. (2024)
miR-130b-3p	AMPK/mTOR	Inhibit ferroptosis by downregulating the AMPK/mTOR pathway	Qi et al. (2023)
miR-182-5p	GPX4/SLC7A11	Downregulate GPX4 and SLC7A11 and activate ferroptosis in renal injury	Jin et al. (2024)
miR-378a-3p	GPX4/SLC7A11	Downregulate GPX4 and SLC7A11 and activate ferroptosis in renal injury	Ding et al. (2020)
miR-27a-3p	SLC7A11	CircBCAR3 downregulates miR-27a-3p to upregulate SLC7A11 to inhibit ferroptosis and oxidative damage	Lu et al. (2021)

## 5 Clinical applications of exosomes in arteriovenous fistula stenosis

Exosomes have shown significant potential for the treatment of vascular stenosis-related diseases. Traditionally, stem cell therapies have operated primarily through paracrine mechanisms, leading to the development of cell-free stem cell therapies that focus on the application of exosomes. As mediators of intercellular communication, exosomes carry various bioactive molecules including proteins, lipids, and RNA. This capability enables them to play crucial roles in promoting tissue repair, regulating immune responses, and facilitating cellular signaling.

### 5.1 Diagnostic application

Exosomes, found in various body fluids, can reflect the state of their parent cells. Their significant advantages include the capability for rapid detection and noninvasive collection of body fluids. Notably, miRNAs within exosomes offer superior sensitivity and specificity compared to circulating miRNAs in blood, as they can be purified to ensure high sensitivity and specificity. A study utilizing microarray analysis compared miRNA expression profiles between stenotic AVF sites and control veins. The results showed that hsa-miR-214-3p, hsa-miR-374B-3p, and hsa-miR30e-3p were upregulated, whereas hsa-miR-4708-3p, hsa-miR-371b-5p, and hsa-miR-3960 were downregulated in primary AVF (Li et al., 2023). These findings provide valuable guidance for the use of exosomes as biomarkers to detect AVF stenosis. Zhao et al. induced VSMC proliferation using angiotensin II and observed that the expressions of KLF5 and cyclin D1 also increased with increased VSMC proliferation, along with miR-146a upregulation (Li and Chen, 2023). They used salvianolic acid B to downregulate miR-146a expression, thereby inhibiting neointimal hyperplasia. miR-146a has been proposed as a biomarker for inflammation and fibrosis (Lv et al., 2016). A study on patients with periodontitis revealed that serum exosomal miR-146a levels were significantly elevated and exhibited a positive correlation with inflammatory factor levels and disease severity. Inflammation is critical in acute coronary syndrome. Serum miR-146a levels are significantly higher in patients with this condition than in healthy controls. miR-21 is also crucial for vascular intimal stenosis. Overexpression studies have shown that miR-21 downregulation reduces intimal formation in rat carotid arteries after angioplasty. miR-21 can inhibit VSMC proliferation and promote apoptosis. Clinically, samples from stenotic AVF sites show high miR-21 expression, which is predominantly localized in the intimal region where fibroblasts are situated (Zhao et al., 2019). Exosomes containing miR-155 induce EC inflammation, leading to endothelial dysfunction. They increase the production of IL-6 and TNF-α and exert their effects through SHIP1 and SOCS1 (Liao et al., 2023).

High-mobility group box 1 (HMGB1) present in exosomes, and they play a pivotal role in neointimal hyperplasia. HMGB1 promotes inflammatory responses through damage-associated molecular patterns and activates immune cells, leading to the generation of IL-1β (Kilari et al., 2019). HMGB1 and IL-1β together can upregulate MMP-1, MMP-3, and MMP-9 expression, which exacerbate vascular wall degradation and remodeling (Jiang K. et al., 2019). Following vascular injury, VSMCs migrate from the intima to the site of injury. During this process, HMGB1 interacts with receptors for advanced glycation end products and TLRs, thereby promoting VSMC proliferation and migration (Yang and Tracey, 2010). This nuclear protein, which circulates via EVs, can be used as a biomarker, offering significant insights into vascular pathology.

### 5.2 Therapeutic applications

Currently, many nanometer-scale particles are used as platforms for the delivery of therapeutic drugs. However, artificially prepared carriers often face issues such as immune rejection and inability to penetrate specific biological barriers. In contrast, exosomes exhibit excellent biocompatibility and possess membrane structures on their surfaces, making them ideal drug carriers. In addition, proteins, nucleic acids, and other drug molecules encapsulated within exosomes demonstrate greater stability. Exosomes can be artificially engineered to encapsulate various types of drugs and are designed with surface antigens that target specific cells or tissues. Common antiproliferative drugs, such as paclitaxel, can be delivered in a targeted manner using exosomes. Exosomes contain a plethora of proangiogenic and antiinflammatory factors that promote angiogenesis and mitigate inflammatory responses following vascular injury. Exosome-releasing stents have been developed to gradually release exosomes fixed to the stent surface, thereby promoting EC regeneration and vascular wall repair. These stents significantly enhance EC migration and proliferation while reducing smooth muscle cell proliferation in animal models. This technology effectively reduces the restenosis rate after stent implantation and accelerates vascular healing (Wang and Khalil, 2018). Researchers have initiated mesenchymal stem cells release with paclitaxel, reporting that the released microvesicles exhibit strong antiproliferative activity (Wang and Khalil, 2018; Inoue et al., 2007).

The therapeutic applications of exosomes encapsulating miRNAs, lncRNAs, and circRNAs have been extensively studied. VSMC-derived exosomes are rich in miR-145, which regulates VSMC function by inhibiting autophagy, thereby reducing VSMC proliferation and migration (Hu et al., 2021). Animal studies on miR-145 transduction have demonstrated its role in phenotypic modulation, converting proliferative VSMCs into a contractile state. This conversion alleviates neointimal hyperplasia and has shown promising results in the treatment of venous graft diseases (Wang et al., 2020a). Zhao et al. loaded an miR-145-5p agomir (miR-145) into injectable and *in situ* self-assembling RAD hydrogels, allowing for the slow and controlled release of miR-145. This method reduced smooth muscle cell migration, promoted the contractile phenotype, and facilitated EC regeneration. Studies using rat vascular injury models revealed that miR-451 upregulation improved intimal thickening by mitigating VSMC damage caused by PDGF-BB and inhibited VSMC proliferation and migration (Nishio et al., 2020). Researchers using the Shexiang Baoxin pill to influence miR-451 expression and regulate PDGF-BB found that miR-451 can also regulate VSMC phenotypic transformation and promote apoptosis to reduce neointimal hyperplasia after vascular injury (Zhang W. et al., 2019). In another study using a mouse tissue-engineered vascular graft model, miR-451 expression was found to be downregulated in graft stenosis (Li et al., 2022). miR-451 downregulation leads to acute proliferation of macrophages and VSMCs, highlighting a direct regulatory relationship between miR-451 and macrophage migration inhibitory factor, suggesting miR-451 as a potential therapeutic target for intimal stenosis. Exosomes derived from hucMSCs are rich in miR-148a, which targets the 3′untranslated region to inhibit SERPINE1, reducing VSMC phenotypic transformation and migration (Hibino et al., 2016). SERPINE1, also known as plasminogen activator inhibitor-1, is a critical factor contributing to IH.

LncRNAs have emerged as significant targets for intervention in neointimal hyperplasia. Silencing lncRNA-p21 induces proliferation while inhibiting the apoptosis of VSMCs and macrophages (Zhang X. et al., 2022). Liang et al. found that the lncRNA Xist functions in VSMCs by interacting with miR-29b-3p and inducing arterial smooth muscle cell apoptosis via the miR-29b-3p/Eln pathway (Wu et al., 2014). Song et al. demonstrated that overexpression of lncRNA SENCR regulates the miR206/myocardin axis, thereby inhibiting VSMC proliferation, migration, and phenotypic transformation (Liang et al., 2021). Cheng et al. identified that the lncRNA LLNC00281 negatively regulates ANXA2 and inhibits VSMC proliferation, migration, and dedifferentiation through the NF-κBp65 signaling pathway (Song et al., 2022). Another study found that the lncRNA RP11-531A24.3 also negatively regulates ANXA2 (Verma Nagendra and Arora, 2025). Similarly, circRNAs have been implicated in IH and are considered potential therapeutic targets. Fu et al. found that circMAPK1 regulates VSMC proliferation and migration via the miR22-3p/MECP2 axis (Cheng et al., 2022). Lin et al. reported that circ_0021155 expression increases ox-LDL-induced VSMC proliferation and migration (Wu et al., 2021). This circRNA promotes TRPM7 expression while inhibiting the expression of α-smooth muscle actin and calmodulin, thereby enhancing smooth muscle cell proliferation and migration.

Exosomes influence neointimal hyperplasia by interfering with ferroptosis. Various miRNAs within exosomes regulate iron and ROS metabolism during ferroptosis, thereby affecting cellular redox states (Lin et al., 2022). Understanding the mechanisms by which miRNAs regulate ferroptosis is crucial for diagnosing and treating IH. Exosomes derived from human umbilical cord blood stem cells can inhibit ferroptosis by suppressing DMT1 expression via miR-23a-3p (Zong et al., 2022). Jia et al. found that exosomes from endothelial progenitor cells upregulate miR-30e-5p in HUVECs, activating the AMPK pathway. miR-30e-5p directly targets and inhibits SP1, thereby preventing EC ferroptosis (Song et al., 2021). Additionally, Li et al. demonstrated that exosomes from endothelial progenitor cells transfer miR-199a-3p to ECs, reducing ROS production and iron accumulation, ultimately inhibiting EC ferroptosis and death (Xia et al., 2022).

However, the use of exosomes as drug carriers has several limitations. First, exosomes derived from stem cells are limited by the manufacturing methods, making large-scale, high-yield production challenging (Li et al., 2021). Second, the loading capacity of exosomes is relatively low, and they are easily captured by non-target organs, thereby reducing their effective dosage. Thus, surface modification of exosomes is crucial to enhance their targeting and efficacy (Pascucci et al., 2014; Colao et al., 2018).

### 5.3 Challenges and limitations in clinical translation

Despite the promising roles of exosomes in regulating vascular remodeling, inflammation, and cell proliferation in AVF stenosis, several critical challenges hinder their clinical translation. First, the heterogeneity of exosome populations derived from different cell types and biofluids complicates standardization and quality control. Secondly, current isolation and purification techniques, such as ultracentrifugation and precipitation, often yield preparations with impurities or low reproducibility, limiting their scalability for clinical-grade production. Moreover, the targeting efficiency and *in vivo* biodistribution of exogenously administered exosomes remain suboptimal, raising concerns about their delivery to pathological sites. In addition, the mechanisms underlying exosome-mediated signaling are not fully elucidated, especially in the context of AVF-specific microenvironments. Regulatory hurdles, lack of large-scale clinical trials, and insufficient understanding of long-term safety further complicate the path to clinical application. Therefore, while exosomes hold great promise, their use in treating AVF stenosis remains at a preclinical stage and requires further technological, mechanistic, and translational advancements (Verma Nagendra and Arora, 2025).

## 6 Conclusion

Exosomes have demonstrated significant potential in the diagnosis and treatment of AVF stenosis. As noninvasive biomarkers, exosomes can be used to detect AVF stenosis at an early stage. They also regulate VSMC function by carrying specific miRNAs and proteins, thereby mitigating inflammatory responses and preventing stenosis progression. Furthermore, exosome-based therapeutic approaches offer an accurate targeted treatment strategy, potentially enhancing therapeutic efficacy and reducing adverse effects. This study reviewed the biogenesis, isolation, and identification of exosomes and their potential applications in AVF stenosis. Despite the growing body of evidence supporting the potential of exosomes in regulating AVF pathophysiology, significant challenges remain before their clinical translation. The lack of standardized isolation protocols, variability in exosomal cargo, and limited *in vivo* validation hinder progress toward therapeutic implementation. In our view, future research should prioritize the development of engineered exosomes with targeted delivery capabilities, integration with biomaterials such as hydrogels for sustained release, and validation using large-scale clinical cohorts. Furthermore, establishing diagnostic panels using exosome-derived biomarkers specific to AVF stenosis could significantly improve early detection and personalized treatment strategies. Addressing these challenges through multidisciplinary approaches will be crucial to unlocking the full clinical utility of exosomes in AVF management.

## Glossary

**AVF**: arteriovenous fistula
circRNA: circular RNA
**DLS**: dynamic light scattering
**DNA**: deoxyribonucleic acid
**EPCs**: endothelial progenitor cells
**ESE**: early sorting endosome
**EVs**: extracellular vesicles
**GPX4**: glutathione peroxidase 4
**HMGB1**: High-mobility group box 1
**hucMSCs**: human umbilical cord mesenchymal stem cells
**HUVECs**: human umbilical vein endothelial cells
**IH**: intimal hyperplasia
**IL**: interleukin
**ILVs**: intracellular vesicles
**lncRNA**: long noncoding RNA
**miRNA**: microRNA
**MMP**: matrix metalloproteinases
**mRNA**: messenger RNA
**MSCs**: mesenchymal stem cells
**MVBs**: multivesicular bodies
**NF-κB**: nuclear factor kappa B
**NO**: nitric oxide
**NTA**: nanoparticle tracking analysis
**PDGF**: platelet-derived growth factor
**PEG**: polyethylene glycol
**PS**: phosphatidylserine
**Re**: Reynolds number
**RNA**: ribonucleic acid
**ROS**: reactive oxygen species
**SEC**: size exclusion chromatography
**SM**: sphingomyelin
**TEM**: transmission electron microscopy
**TLRs**: toll-like receptors
**TNF-α**: tumor necrosis factor alpha
**VE-cadherin**: vascular endothelial cadherin
**VEGF**: vascular endothelial growth factor
**VSMC**: vascular smooth muscle cells
**ZnONP**: ZnO nanoparticles
